# Neural Correlates of Disgust Processing in Childhood Maltreatment and Peer Victimisation

**DOI:** 10.1192/bjo.2024.193

**Published:** 2024-08-01

**Authors:** Lena Lim

**Affiliations:** SICS, ASTAR, Singapore, Singapore. IoPPN, King's College London, London, United Kingdom

## Abstract

**Aims:**

Childhood maltreatment (CM) and peer victimisation (PV) are common sources of early-life interpersonal stress. CM is associated with atypical fronto-limbic emotion processing and regulation, and increased vulnerability for self-harm/suicide. However, few studies have compared the neurofunctional correlates between caregiver-inflicted versus peer-inflicted mistreatment. We compared the alterations of neurofunctional correlates of facial emotion processing in young people exposed to CM or PV and explored their associations with self-harm.

**Methods:**

fMRI data were collected from 114 age- and gender-matched youths (39 CM, 37 PV and 38 controls) during an emotion discrimination task. Region-of-interest (amygdala, insula) and whole-brain analyses were conducted.

**Results:**

Groups differed significantly during processing of disgust only. Both CM and PV groups had lower activation in right amygdala and bilateral posterior insula than controls, where the left insular underactivation was furthermore related to increased self-harm in maltreated youths. At the whole-brain level, both CM and PV groups also had underactivation compared with controls in a cluster of bilateral limbic-thalamic-striatal, precuneus/posterior cingulate, temporal, fusiform/lingual and cerebellar regions, which was negatively associated with emotional problems in controls, as well as a cluster of somatosensory regions associated with increased self-harm in maltreated youths.

**Conclusion:**

Early-life interpersonal stress from caregivers or peers is associated with common underactivation of limbic-thalamic-striatal, precuneus/posterior cingulate and somatosensory regions during disgust processing. The hypoactivation of key emotion and sensory processing and self-referential brain regions could be a potential suppressive mechanism to cope with the aversive emotion; however, it may also entail increased risk of affective psychopathology in seemingly healthy youths.

